# A survey of parasitoids from Greece with new associations

**DOI:** 10.3897/zookeys.817.30119

**Published:** 2019-01-15

**Authors:** Nickolas G. Kavallieratos, Saša S. tanković, Martin Schwarz, Eleftherios Alissandrakis, Christos G. Athanassiou, George D. Floros, Vladimir Žikić

**Affiliations:** 1 Laboratory of Agricultural Zoology and Entomology, Department of Crop Science, Agricultural University of Athens, 75 Iera Odos str., 11855, Athens, Attica, Greece Agricultural University of Athens Athens Greece; 2 Faculty of Sciences and Mathematics, Department of Biology and Ecology, University of Niš, Višegradska 33, 18000, Niš, Serbia University of Niš Niš Serbia; 3 Biologiezentrum, Johann Wilhelm Klein Straße 73, 4040, Linz, Austria Biologiezentrum Linz Austria; 4 Laboratory of Entomology and Pesticide Science, Department of Agriculture, Technological Educational Institute of Crete, P.O. Box 1939, 71004, Heraklion, Crete, Greece Technological Educational Institute of Crete Heraklion Greece; 5 Laboratory of Entomology and Agricultural Zoology, Department of Agriculture, Crop Production and Rural Environment, University of Thessaly, Phytokou Street, 38446, Nea Ionia, Magnissia, Greece University of Thessaly Nea Ionia Greece; 6 Laboratory of Applied Zoology and Parasitology, School of Agriculture, Aristotle University of Thessaloniki, 54124, Thessaloniki, Greece Aristotle University of Thessaloniki Thessaloniki Greece

**Keywords:** Balkans, field pests, natural enemies, urban pests

## Abstract

We report 22 parasitoid species from Greece that have emerged from their hosts belonging to Blattodea, Coleoptera, Hymenoptera and Lepidoptera, including 12 Braconidae, one Eulophidae, one Evaniidae, seven Ichneumonidae, and one Tachinidae. Nine parasitoids are reported for the first time in Greece, i.e., three Ichneumonidae: *Campoplexdifformis* (Gmelin, 1790), *Gelisalbipalpus* (Thomson, 1884), and *Lysibiatenax* Townes, 1983; five Braconidae: *Charmoncruentatus* Haliday, 1833, *Dendrosoterprotuberans* (Nees, 1834), *Dolichogenidealongipalpis* (Reinhard, 1880), *Ecphylussilesiacus* (Ratzeburg, 1848), and *Spathiuscurvicaudis* Ratzeburg, 1844; and one Eulophidae: *Melittobiaacasta* (Walker, 1839). Nine of the 23 recorded parasitoid-host associations are new. These findings are discussed in relation to the overall related parasitoid-host associations in the target area, as well as the potential of parasitoid use in the biological control of pests.

## Introduction

Parasitoids, especially those belonging to Hymenoptera, are important elements of agroecosystems (Godfray 1994). Faunistic surveys of parasitoids constitute the major baselines upon which further applicative studies are based (Tomanović et al. 2014; Petrović et al. 2019). Many pest species, especially lepidopterans, are prone to increase their abundance if conditions are favorable and inflict significant hazards in agriculture and forestry (Elkinton and Liebhold 1990; Devetak et al. 2014). As many parasitoids are able to attack several host species or to specialize on a certain host species, their population dynamics depends on their hosts’ abundance, and thus, they are of major importance in controlling pests (Hassell 1980; Hassell and May 1986; Barbosa 1998; Gagić et al. 2016). Systematic investigations of parasitoid fauna in Greece, except for Aphidiinae (Hymenoptera: Braconidae) (Kavallieratos et al. 2004, 2006, 2008, 2013, 2016), have scarcely been conducted. In fact, some surveys of the parasitoid spectrum in Greece in various host species, with the exception of Aphidiinae, have been initiated only during the last years. For example, Kolarov (2007) catalogued the Ichneumonidae (Hymenoptera) of Greece, Papp (2007) presented a list of Braconidae, and Zeegers (2017) provided information on some Tachinidae (Diptera). Other studies are mostly related to particular subgroups of parasitoids. For instance, Tsankov et al. (1999) reported egg parasitoids of *Thaumetopoeapityocampa* (Denis & Schiffermüller, 1775) (Lepidoptera: Notodontidae), Anagnou Veroniki et al. (2006) studied the parasitization of *Phyllocnistiscitrella* Stainton, 1856 (Lepidoptera: Gracillariidae), Žikić et al. (2017) revealed the parasitoids of two serious pests in forestry, *Lymantriadispar* (L., 1758) (Lepidoptera: Erebidae) and *Malacosomaneustria* (L., 1758) (Lepidoptera: Lasiocampidae), and Alissandrakis et al. (2018) investigated the parasitoid complex that is related to *Praysoleae* (Bernard, 1788) (Lepidoptera: Praydidae). Considering the lack of information on the spectrum of parasitoids of noxious insects in Greece, the objective of our study was to conduct a survey and shed light on the associations between hymenopterous or dipterous parasitoids and their field or urban hosts in the country.

## Materials and methods

**Figure 1.**
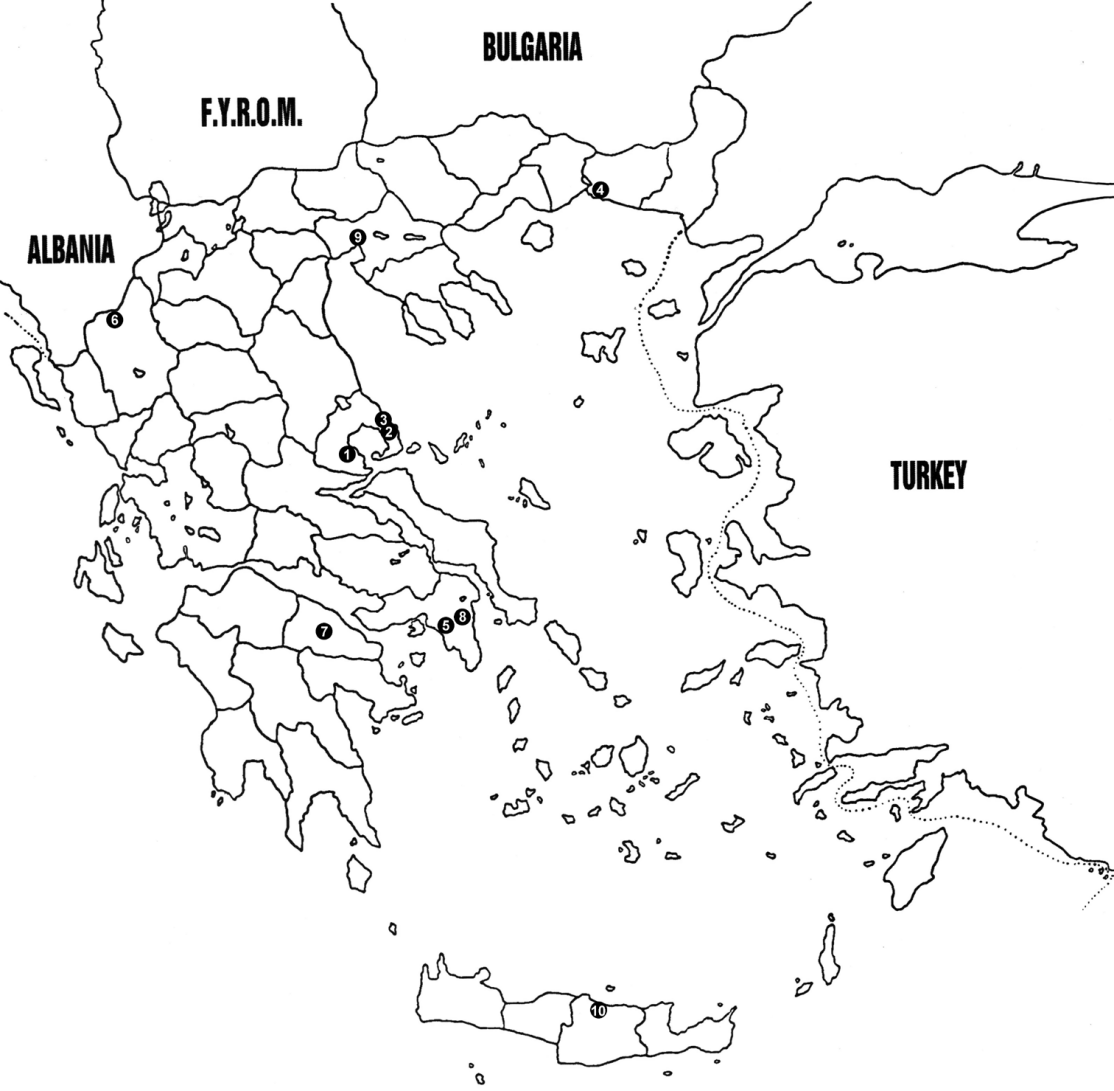
Investigated areas marked on the map of Greece: **1** Almyros (Thessaly) **2** Agios Ioannis (Thessaly) **3** Agrioleukes (Thessaly) **4** Arogi (Thrace) **5** Athens (central Greece) **6** Grammos (Epirus) **7** Nemea (Peloponnese) **8** Tatoi (central Greece) **9** Thessaloniki (Macedonia) **10** Voutes (Crete).

The samples were collected in several localities on the Greek mainland (central Greece, Epirus, Macedonia, Peloponnese, Thrace) and the island of Crete (Fig. 1). The insect material was collected either with or without visible signs of parasitism. Insect specimens were collected from field or urban environments, and then separately placed in plastic containers covered with nylon mesh, transferred to the laboratory and reared at 25 °C until parasitoid emergence. When host insects were found on plants, voucher samples of the plants were kept in herbariums and later identified by Prof. Bojan Zlatković (Department of Biology and Ecology, University of Niš). To collect specimens of *Ptosimaundecimmaculata* (Herbst, 1784) (Coleoptera: Buprestidae) and *Scolytusrugulosus* (Müller, 1818) (Coleoptera: Curculionidae), infested wood was cut into small pieces. Then, larvae were very carefully removed, put in plastic containers and kept in the same conditions as above. As soon as the adult parasitoids were emerged, they were captured using an aspirator and killed into vials containing 70% ethyl alcohol. They were then slide-mounted for detailed examination. Specimens for slides were washed in distilled water, boiled in 10% KOH for 2 minutes, rewashed, and then placed in a drop of Faure-Berlese medium (Krantz 1978) for dissection or whole mounting. External morphology was studied using an Olympus SZX9 (zoom ratio 9; total magnification 12.6–114.0×) (Olympus Corporation, Tokyo, Japan) or a Zeiss Discovery V8 (zoom ratio 8; total magnification 10.0–80.0×) (Carl Zeiss MicroImaging GmbH, Göttingen, Germany) stereomicroscopes. The identification of parasitoids was based on Tobias and Jakimavicius (1986), Tschorsnig and Herting (1994), Broad (2011), while the host identifications were conducted by Prof. Josef J. De Freina (Museum Witt, Munich, Germany) on the basis of larval morphology. The material was deposited in the insect collection of the Laboratory of Agricultural Zoology of Entomology, Agricultural University of Athens, Greece, the Laboratory of Entomology, Department of Agriculture, Technological Educational Institute of Crete, Greece, and the Faculty of Sciences and Mathematics, Department of Biology and Ecology, University of Niš, Serbia. Nomenclature of hosts is based on Goulet and Huber (1993). In the following results, new records of parasitoids for Greece are marked with an asterisk (*) in front of each parasitoid name while new parasitoid-host associations are indicated with a dagger (†) in front of each host name. Dates indicate the time when the hosts were collected.

## Results

The identifications of adult parasitoids which emerged from their hosts, revealed 22 species belonging to 5 families. The most numerous were those of the family Braconidae (12 species) followed by Ichneumonidae (7 species). Twenty-three parasitoid-host associations, nine of which were not previously known, are listed in detail below. Nine parasitoid species are recorded for the first time from Greece.

### Family Braconidae

#### Subfamily Brachistinae

***Triaspisthoracica* (Curtis, 1860)**

**Material examined.** 13♀, 18♂, Macedonia, Thessaloniki (40°37'58.07"N, 22°57'28.47"E), 01 March 2016, leg. D. Koveos. Host: *Bruchuspisorum* (L., 1758) (Coleoptera: Chrysomelidae) on *Viciasativa*; 31♀, 13♂, Macedonia, Thessaloniki (40°37'58.07"N, 22°57'28.47"E), 01 March 2016, leg. D. Koveos. Host: *Bruchuspisorum* (L., 1758) (Coleoptera: Chrysomelidae) on *Viciaperegrina*.

#### Subfamily Charmontinae

* ***Charmoncruentatus* Haliday, 1833**

**Material examined.** 2♀, Thessaly, Almyros (39°10'34.39"N, 22°45'17.46"E), 11 May 2016, leg. S. Stanković. Host: †*Tortrixviridana* L., 1758 (Lepidoptera: Tortricidae) on *Quercuspubescens*.

#### Subfamily Cheloninae

**Chelonus (Microchelonus) sulcatus Jurine, 1807**

**Material examined.** 2♀, 1♂, Macedonia, Thessaloniki (40°37'55.03"N, 22°57'25.57"E), 15 July 2002, leg. N. G. Kavallieratos. Host: *Prayscitri* (Millière, 1873) (Lepidoptera: Praydidae) on *Citrussinensis*.

#### Subfamily Doryctinae

* ***Dendrosoterprotuberans* (Nees, 1834)**

**Material examined.** 1♀, 3♂, Crete, Heraklion, Voutes (35°15'57.86"N, 25°3'17.11"E), 24 April 2017, leg. E. Alissandrakis. Host: †*Ptosimaundecimmaculata* (Herbst, 1784) (Coleoptera: Buprestidae) on *Prunusdomestica*.

* ***Ecphylussilesiacus* (Ratzeburg, 1848)**

**Material examined.** 2♂, 2♀, Crete, Heraklion, Voutes (35°15'55.58"N, 25°3'42.82"E), 23 May 2017, leg. E. Alissandrakis. Host: *Scolytusrugulosus* (Müller, 1818) (Coleoptera: Curculionidae) on *Prunusdomestica*.

* ***Spathiuscurvicaudis* Ratzeburg, 1844**

**Material examined.** 2♂, 5♀, Crete, Heraklion, Voutes (35°16'8.13"N, 25°3'49.67"E), 16–22 May 2017, leg. E. Alissandrakis. Host: †*Scolytusrugulosus* (Müller, 1818) (Coleoptera: Curculionidae) on *Prunusdomestica*.

#### Subfamily Microgastrinae

***Cotesiaspuria* (Wesmael, 1837)**

**Material examined.** 13♀, 5♂, central Greece, Dekeleia, Tatoi (38°11'59.02"N, 23°47'40.44"E), 07 May 2016, leg. N. G. Kavallieratos, V. Žikić. Host: *Dilobacaeruleocephala* (L., 1758) (Lepidoptera: Noctuidae) on *Pyrusspinosa*.

***Cotesiazygaenarum* Marshall, 1885**

**Material examined.** 7♀, 4♂, Peloponnese, Nemea (37°50'14.26"N, 22°38'37.33"E), 24 May 2016, leg. A. Nahirnić. Host: *Zygaenalonicerae* (Scheven, 1777) (Lepidoptera: Zygaenidae) on *Tetragonolobuspurpureus*.

***Diolcogasteralvearia* (F., 1798)**

**Material examined.** 20♀, 7♂, Thrace, Arogi (40°57'20.77"N, 25°10'12.08"E), 05 June 2005, leg. V. Žikić. Host: *Peribatodesrhomboidaria* (Denis & Schiffermüller, 1775) (Lepidoptera: Geometridae) on *Maluspumila*; 31♀, 8♂, Athens (37°58'54.65"N, 23°44'49.09"E), 07 May 2016, leg. V. Žikić. Host: *Peribatodesrhomboidaria* (Denis & Schiffermüller, 1775) (Lepidoptera: Geometridae) on *Lonicerapileata*.

***Dolichogenideacandidata* (Haliday, 1834)**

**Material examined.** 4♀, Thessaly, Mt Pelion, Agios Ioannis (39°24'58.81"E, 23°9'34.27"E), 10 August 2017, leg. V. Žikić. Host: *Choreutisnemorana* (Hübner, 1799) (Lepidoptera: Choreutidae) on *Ficuscarica*.

* ***Dolichogenidealongipalpis* (Reinhard, 1880)**

**Material examined.** 3♀, 1♂, Epirus, Mt Grammos (40°21'5.95"N, 20°46'43.45"E), 17 July 2003, leg. N. G. Kavallieratos. Host: †*Dahlica* sp. Enderlein, 1912 (Lepidoptera: Psychidae) on rock.

***Glyptapantelesvitripennis* (Curtis, 1830)**

**Material examined.** 3♂, central Greece, Dekeleia, Tatoi (38°9'46.13"N, 23°47'39.34"E), 07 May 2016, leg. N. G. Kavallieratos, V. Žikić. Host: *Dilobacaeruleocephala* (L., 1758) (Lepidoptera: Noctuidae) on *Pyrusspinosa*.

### Family Eulophidae

#### Subfamily Tetrastichinae

* ***Melittobiaacasta* (Walker, 1839)**

**Material examined.** 5♀, central Greece, Dekeleia, Tatoi (38°12'2.17"N, 23°39'53.51"E), 07 May 2016, leg. V. Žikić, N. G. Kavallieratos. Host: †*Parocneriaterebinthi* (Freyer, 1838) (Lepidoptera: Erebidae) on *Pistaciaterebinthus*.

### Family Evaniidae

***Prosevaniafuscipes* (Illiger, 1807)**

**Material examined.** 3♀, 1♂, Thrace, Arogi (40°57'18.70"N, 25°10'6.79"E), 05 August 2016, leg. V. Žikić. Host: *Blattaorientalis* L., 1758 (Blattodea: Blattidae).

### Family Ichneumonidae

#### Subfamily Banchinae

***Lissonotaculiciformis* Gravenhorst, 1829**

**Material examined.** 4♂, central Greece, Dekeleia, Tatoi (38°10'2.26"N, 23°48'15.65"E), 07 May 2016, leg. N. G. Kavallieratos, V. Žikić. Host: *Malacosomaneustria* (L., 1758) (Lepidoptera: Lasiocampidae) on *Quercuscoccifera*.

#### Subfamily Campopleginae

*Campoplexdifformis (Gmelin, 1790)

**Material examined.** 5♀, central Greece, Dekeleia, Tatoi (38°9'53.71"N, 23°49'11.51"E), 07 May 2016, leg. N. G. Kavallieratos, V. Žikić. Host: †*Pammeneoxycedrana* (Millière, 1876) (Lepidoptera: Tortricidae) on *Arbutusunedo*; 1♀, central Greece, Dekeleia, Tatoi (38°10'2.98"N, 23°49'40.93"E), 07 May 2016, leg. N. G. Kavallieratos, V. Žikić. Host: *Archipsrosana* (L., 1758) (Lepidoptera: Tortricidae) on *Pyrusspinosa*.

#### Subfamily Cryptinae

*Gelisalbipalpus (Thomson, 1884)

**Material examined.** 6♀, Thessaly, Mt Pelion, Agrioleukes (39°23'13.74"N, 23°5'1.00"E), 11 May 2016, leg. S. Stanković, V. Žikić. Host: †*Cotesianeustriae* (Tobias, 1986) (Hymenoptera: Braconidae) parasitizing *Lymantriadispar* (L., 1758) (Lepidoptera: Erebidae) feeding on *Quercuscoccifera*.

***Gelisareator* (Panzer, 1804)**

**Material examined.** 3♀, 1♂, Thessaly, Mt Pelion, Agrioleukes (39°23'15.16"N, 23°5'1.36"E), 11 May 2016, leg. S. Stanković, V. Žikić. Host: †*Cotesianeustriae* (Tobias, 1986) parasitizing *Lymantriadispar* (L., 1758) (Lepidoptera: Erebidae) feeding on *Quercuscoccifera*.

***Gelisilicicola* (Seyrig, 1927)**

**Material examined.** 4♀, Thessaly, Mt Pelion, Agrioleukes (39°23'16.90"N, 23°5'3.91"E), 11 May 2016, leg. S. Stanković, V. Žikić. Host: *Cotesianeustriae* (Tobias, 1986) parasitizing *Lymantriadispar* (L., 1758) (Lepidoptera: Erebidae) feeding on *Quercuscoccifera*.

*Lysibiatenax Townes, 1983

**Material examined.** 12♀, 9♂, central Greece, Dekeleia, Tatoi (38°10'53.42"N, 23°46'50.76"E), 07 May 2016, leg. N. G. Kavallieratos, V. Žikić. Host: †*Cotesianeustriae* (Tobias, 1986) parasitizing *Lymantriadispar* (L., 1758) (Lepidoptera: Erebidae) feeding on *Quercuscoccifera* L.

#### Subfamily Pimplinae

***Itoplectistunetana* (Schmiedeknecht, 1914)**

**Material examined.** 6♀, Thessaly, Mt Pelion, Agios Ioannis (39°25'9.74"N, 23°9'21.43"E), 10 August 2017, leg. V. Žikić. Host: *Choreutisnemorana* (Hübner, 1799) (Lepidoptera: Choreutidae) on *Ficuscarica*.

### Family Tachinidae

#### Subfamily Exoristinae

***Exoristasegregata* (Rondani, 1859)**

**Material examined.** 4♂, central Greece, Dekeleia, Tatoi (38°9'23.16"N, 23°45'3.40"E), 07 May 2016, leg. N. G. Kavallieratos, V. Žikić. Host: *Parocneriaterebinthi* (Freyer, 1838) (Lepidoptera: Erebidae) on *Pistaciaterebinthus*.

## Discussion

Our findings revealed that the subfamily Charmontinae is represented by *C.cruentatus*, which is a solitary parasitoid of several microlepidopterans (van Achterberg 1979; Yu et al. 2012) and is recorded in Greece for the first time. The only identified species of the subfamily Cheloninae, *C.sulcatus*, is a parasitoid of several microlepidopterous insects (Aydogdu and Beyarslan 2006). It is an important natural enemy of *Prayscitri* (Millière, 1873) (Lepidoptera: Praydidae), a pest of citrus that is common in the Mediterranean region (Moreno et al. 1990). Most likely, this moth has been introduced in the Mediterranean region from Africa with the import of citrus propagating material (de Carvalho and Aguiar 1997). The evaluation of *C.sulcatus* as a biological control agent against *P.citri* could be of interest, given that this parasitoid species is widely spread in the Palaearctic (Aydogdu and Beyarslan 2006).

One of the largest braconid subfamily, Microgastrinae, is specialized on the parasitization of caterpillars (Shaw and Huddleston 1991). Both identified species of *Cotesia* Cameron (*C.spuria* and *C.zygaenarum*) are gregarious endoparasitoids of caterpillars (Žikić et al. 2013; Gadallah et al. 2015). *Cotesiaspuria* is a polyphagous and cosmopolitan species that inhabits whole Palaearctic (Yu et al. 2012; Gadallah et al. 2015). During our study we identified *C.spuria* parasitizing caterpillars of *D.caeruleocephala*, which is recognized as a pest in orchards of the family Rosaceae (Bolu and Özgen 2007). *Cotesiazygaenarum* is an oligophagous parasitoid of the genus *Zygaena* F. and several other lepidopterous genera (Gadallah et al. 2015). Another gregarious parasitoid identified in this study, *D.alvearia*, has a very narrow host range which includes 9 taxa of coleopterous, hymenopterous and lepidopterous insects commonly found in Europe (Gadallah et al. 2015). The controversial genus *Dolichogenidea* Viereck, which is hardly separated from *Apanteles* Foerster (sensu stricto) (Fernández Triana et al. 2014), predominantly contains both solitary and gregarious endoparasitoids of microlepidoptera (i.e., Gracillariidae, Plutellidae, Pyralidae, Tortricidae, Yponomeutidae) (Medvedev 1989; Jankowska and Wiech 2006). *Glyptapantelesvitripennis* is a gregarious parasitoid with broad host range that includes major pests in forest and agricultural ecosystems, e.g., *L.dispar*, *M.neustria*, *Yponomeutamalinellus* Zeller, 1838 (Lepidoptera: Yponomeutidae) (Yu et al. 2012). Interestingly, this species emerged from *D.caeruleocephala* caterpillars under no evident superparasitism because some other caterpillars were parasitized by *C.spuria*.

The subfamily Doryctinae includes species that parasitize wood-feeding coleopterans, including Curculionidae (Scolytinae), Bostrychidae, Buprestidae and Cerambycidae (Farahani et al. 2014). *Ptosimaundecimmaculata* is identified for the first time as a host of *D.protuberans* in our study. Larvae of *P.undecimmaculata* live inside wood of dead trees and living branches of different species of *Prunus* spp. for 2–3 years (Sakalian 2003). Moreover, with the exception of *Dolichomitustuberculatus* (Geoffroy, 1785) (Hymenoptera: Ichneumonidae), parasitoids of this species have not been previously recorded (Aliyev and Maharramova 2009). *Dendrosoterprotuberans* is an oligophagous ectoparasitoid recorded from numerous hosts, mainly Curculionidae, e.g., *Hylesinus* F., *Leperisinus* Reitter, *Phloeosinus* Chapuis, *Phloeotribus* Latreille, *Scolytus* Geoffroy, *Tomicus* Latreille, and also from few Buprestidae, Cerambycidae and Chrysomelidae (Wegensteiner et al. 2015; Beyarslan 2017). This parasitoid species is a very important natural enemy of *Scolytusmultistriatus* (Marsham, 1802) (Coleoptera: Curculionidae) that seriously attacks elm trees in Europe (Manojlović et al. 2003). The genus *Ptosima* Solier is solely represented in Europe by *P.undecimmaculata* with two subspecies; Ptosimaundecimmaculatassp.metallescens Bily, 1982 (Coleoptera: Buprestidae) which is only found in Crete, and Ptosimaundecimmaculatassp.undecimmaculata (Herbst, 1784) (Coleoptera: Buprestidae) which is dispersed in the rest of Greece and Europe (van Achterberg 2013). Besides *D.protuberans*, two more species of the genus *Dendrosoter* Wesmael have been reported in Europe, i.e., *Dendrosotercurtisii* (Ratzeburg, 1848) (Coleoptera: Buprestidae) and *Dendrosotermiddendorffii* (Ratzeburg, 1848) (Coleoptera: Buprestidae) (van Achterberg 2013), which have been supplemented by *E.silesiacus* and *S.curvicaudis* as new members of the Greek fauna recorded in our study. The fact that *S.curvicaudis* is recorded for the first time from larvae of *S.rugulosus* in cut woods of *P.domestica* may open a new path towards the biological control of this serious scolytid pest of fruit and nut trees (Seybold et al. 2016), whose chemical control is not effective when *S.rugulosus* is inside wood (Fox 2015).

With few exceptions, *T.thoracica* is a specialized parasitoid of the genus *Bruchus* L., which includes various species of economic importance for stored legumes (Parker 1957; Reddy et al. 2018). This species belongs to the subfamily Brachistinae that includes endoparasitoids of various beetles (Belokobylskij 1998). Several species oviposit inside eggs and emerge from larvae of their hosts, becoming therefore egg-larval parasitoids (Shaw and Huddleston 1991). *Triaspisthoracica* acts that way leading to successful biological control of the noxious *B.pisorum* (Clausen 1954; Nikolova 2016).

Out of the five ensign wasp species inhabiting Europe, four are recorded in Greece, these are: *Evaniaappendigaster* (L., 1758), *P.fuscipes*, *Prosevaniaincerta* (Kieffer, 1904) and *Zeuxevaniasplendidula* (Costa, 1884) (van Achterberg 2013). These wasps are specialized parasitoids of oothecae of Blattodea (Cameron 1957; Carlson 1979). *Prosevaniafuscipes* has been recorded in several European countries, but most likely it occurs in all countries where its host, *B.orientalis*, is present (Ceianu 1986). The only identified member of the family Eulophidae, *M.acasta*, is a gregarious ectoparasitoid of insects belonging to various orders (Browne 1922) (i.e., Hymenoptera, Diptera, Lepidoptera and Coleoptera); it was reared from *P.terebinthi*, which constitutes a new host record.

We also identified seven parasitoid species that belong to the largest hymenopterous family, the Ichneumonidae. *Itoplectistunetana* is primary endoparasitoid of lepidopterous larvae or pupae, while *C.difformis* and *L.culiciformis* emerge from lepidopterous larvae (Georgiev 2000; Talebi et al. 2005; Yu et al. 2012; Žikić et al. 2017). *Lysibiatenax* is a specialized hyperparasitoid of the genus *Cotesia*, while *C.neustriae* is a newly recorded host for this species. The remaining three ichneumonids belong to genus *Gelis* Thunberg and behave either as primary parasitoids or hyperparasitoids; this type of strategy is usual for most of the members of this genus (Žikić et al. 2017). As a secondary parasitoid, *G.albipalpus* has been recorded only from *Apantelesmurinanae* Čapek & Zwölfer, 1957 (Hymenoptera: Braconidae) (Čapek and Zwölfer 1957), and therefore, *C.neustriae* is listed for the first time as a host. The polyphagous *G.aerator*, having a host range that exceeds 180 species (Yu et al. 2012), was found for first time parasitizing also *C.neustriae*. Out of about 25 braconid species on the list of hosts of *G.aerator*, there are 12 species of the genus *Cotesia* (Yu et al. 2012). Prior to the revision by Schwarz (2016) of the macropterous females of genus *Gelis* from the Western Palaearctic, *G.ilicicola* was often mixed with related species and thus most records are not reliable. However, Schwarz (2016) reported that specimens of *G.ilicicola* reared from small lepidopterans (Coleophoridae), neuropterans (Chrysopidae) and hymenopterans (Braconidae: Microgastrinae), suggesting that this species is a parasitoid of a wide range of small cocoons and cocoon-like structures. This hypothesis posits additional investigation provided that the ability of a parasitoid to attack the immobile life stages of host species may have some advantages for its use in biological control. For example, in several cases, larvae choose a site to pupate other than the original feeding one, where they are parasitized by pupal parasitoids (Hawkins 1994; Cancino et al. 2012). In this way, pupal parasitoids restrict the suitable locations for insects’ development (Machtinger et al. 2015).

The only identified species of the family Tachinidae in our study is *E.segregata*. This is a common parasitoid of numerous lepidopterous species (Mückstein et al. 2007). Until now, it has been reared from over 50 species belonging to the Erebidae, such as *L.dispar*, and the Lasiocampidae, such as *M.neustria*, but also from many belonging to the Noctuidae, Thaumetopoeidae and Zygaenidae (Tschorsnig 2017, Žikić et al. 2017). *Exoristasegregata* has a relatively wide geographical range, and it has been recorded in most European countries but also in the North Africa, Anatolia and the USA (Tschorsnig 1997).

Our findings shed light on a greatly overlooked issue, which is the fauna of parasitoids occurring in Greece. The several new recorded parasitoid species in Greece and the previously unknown parasitoid-host associations indicate the high level of biodiversity that exists in the investigated ecosystems and potentially triggers natural biological control of several harmful insect species. The recorded elevated richness of beneficial insects makes us hypothesize that additional natural enemies may occur in the same or similar ecosystems, a premise that should be further investigated and also confirmed with additional field surveys. Furthermore, a common practice of pests' management deals with their identification and the application of chemical compounds without considering the role of the existing beneficial fauna (Mehle and Trdan 2012). Our study clearly shows that the overlooked intermediate stage of identification of parasitoids is also crucial, as it can bring to light the high level of activity of these species against pests. More carefully designed pest management strategies would be modelled towards the conservation of the local parasitoid species.
